# Human Immunodeficiency Virus-1 Viral Load Is Elevated in Individuals With Reverse-Transcriptase Mutation M184V/I During Virological Failure of First-Line Antiretroviral Therapy and Is Associated With Compensatory Mutation L74I

**DOI:** 10.1093/infdis/jiz631

**Published:** 2019-11-27

**Authors:** J Gregson, S Y Rhee, R Datir, D Pillay, C F Perno, A Derache, R S Shafer, R K Gupta

**Affiliations:** 1 Department of Biostatistics, London School of Hygiene and Tropical Medicine, London, United Kingdom; 2 Department of Medicine, Stanford University, Stanford, California, USA; 3 Division of Infection and Immunity, UCL, London, United Kingdom; 4 Africa Health Research Institute, Durban, South Africa; 5 Department of Oncology and Haematoncology, University of Milan, Milan, Italy; 6 Department of Medicine, University of Cambridge, Cambridge, United Kingdom

**Keywords:** antiretroviral, compensatory mutation, drug resistance, HIV, lamivudine

## Abstract

**Background:**

M184V/I cause high-level lamivudine (3TC) and emtricitabine (FTC) resistance and increased tenofovir disoproxil fumarate (TDF) susceptibility. Nonetheless, 3TC and FTC (collectively referred to as XTC) appear to retain modest activity against human immunodeficiency virus-1 with these mutations possibly as a result of reduced replication capacity. In this study, we determined how M184V/I impacts virus load (VL) in patients failing therapy on a TDF/XTC plus nonnucleoside reverse-transcriptase inhibitor (NNRTI)-containing regimen.

**Methods:**

We compared VL in the absence and presence of M184V/I across studies using random effects meta-analysis. The effect of mutations on virus reverse-transcriptase activity and infectiousness was analyzed in vitro.

**Results:**

M184I/V was present in 817 (56.5%) of 1445 individuals with virologic failure (VF). Virus load was similar in individuals with or without M184I/V (difference in log_10_ VL, 0.18; 95% confidence interval, .05–.31). CD4 count was lower both at initiation of antiretroviral therapy and at VF in participants who went on to develop M184V/I. L74I was present in 10.2% of persons with M184V/I but absent in persons without M184V/I (*P* < .0001). In vitro, L74I compensated for defective replication of M184V-mutated virus.

**Conclusions:**

Virus loads were similar in persons with and without M184V/I during VF on a TDF/XTC/NNRTI-containing regimen. Therefore, we did not find evidence for a benefit of XTC in the context of first-line failure on this combination.

## Teaser

Lamivudine is a cornerstone antiretroviral whose efficacy has been ascribed to high fitness cost of the lamivudine resistance mutation M184V. However, here we demonstrate elevated viral loads in the context of M184V, likely attributable to compensatory mutations such as L74I.

**(See the Editorial Commentary by Kuritzkes, on pages 1067–9.)**

The global scale up of antiretroviral therapy (ART) using a public health approach with limited viral load (VL) monitoring has been accompanied by high prevalence of drug resistance to nonnucleoside reverse-transcriptase inhibitor (NNRTI)-containing regimens among individuals with virological failure (VF) in low- and middle-income countries (LMICs) [1–6].

The cytosine analogs lamivudine (3TC) and emtricitabine (FTC), collectively referred to as XTC, are components of first- and second-line regimens recommended by the World Health Organization (WHO). However, high-level XTC resistance can be conferred and selected by single amino acid changes at position 184 of reverse transcriptase (RT) in the highly conserved (Y183, M184, D185, D186) amino acid domain that includes the active (catalytic) site of the p66 polymerase subunit of RT [7]. M184V/I are the most commonly occurring drug-resistant mutations in persons with acquired resistance to first-generation NNRTI-containing regimens [1–6].

Several lines of evidence suggest that in addition to causing high-level reductions in XTC susceptibility in vitro and modestly increased tenofovir disoproxil fumarate (TDF) susceptibility, viruses with these mutations retain some in vivo susceptibility to XTC possibly because of their reduced replication capacity [8–10]. For example, early studies showed that in patients receiving 3TC monotherapy, or dual therapy with AZT/3TC, VL did not return to baseline despite the development of M184V [9, 11–14]. In addition, discontinuation of 3TC during combination ART (cART) was associated with a modest increase in VL [15–17]. By contrast, the COLATE study, a randomized controlled trial conducted in Europe in the early 2000s, showed that there was no effect of removal of 3TC from a failing regimen in which the endpoint was viral suppression to <200 copies/mL or VL change of 1.4 log_10_ [18].

Therefore, to understand the relationship between M184I/V and VL in the era of tenofovir-based cART in which thymidine analog mutations (TAMs) were not present, and also in the context of limited or no access to VL monitoring, we studied individuals failing the WHO-recommended first-line regimen of TDF/XTC/NNRTI across a range of settings [19].

## METHODS

The study population has previously been described and is presented in Supplementary Table 1 [20–41]. Patients treated with TDF plus 3TC/FTC and nevirapine/efavirenz (EFV) were included when documented VF and RT sequence data from codons 40 to 240 were available. Virologic failure was locally determined, and the threshold for LMICs was 1000 copies/mL. Human immunodeficiency virus (HIV)-1 RT sequences were determined by standard Sanger sequencing at individual study sites.

Mutations were defined as amino acid differences at positions 1 to 240 between each sequence and the consensus subtype B amino acid reference sequence. Because some individuals may have been exposed to thymidine analogs before TDF-containing regimens [5], we excluded individuals with sequences containing TAMs—M41L, D67N, K70R, L210W, T215Y/F, and K219Q/E.

Each sequence was subtyped as previously described, and sequence quality control measures were taken to identify sequences with APOBEC (apolipoprotein B mRNA editing catalytic polypeptide-like) G-to-A hypermutation [20]. Duplicate sequences were removed. All patients reported that they were *antiretroviral (*ARV) naive at baseline. The primary outcome was VL at VF; hence, patients without this outcome were excluded.

### Statistical Analysis

We graphically compared the distribution of log_10_ VLs according to presence of M184I/V mutation both within and across studies. To quantify the impact of M184I/V on VL, we calculated mean log_10_ VL in each study according to M184I/V. Differences were pooled across studies using random-effects meta-analysis. Estimates of the standard error in each study were calculated by dividing the pooled estimate of the standard deviation by the square root of the number of patients with/without M184I/V in any given study. We repeated this process in subgroups of patients defined by several baseline characteristics: presence of K65R mutation, presence of major NNRTI mutations, choice of NRTI, choice of NNRTI, categories of baseline CD4 count (< and >200 cells/mm^3^), and categories of baseline VL (< and >100 000 copies per mL). We used the same methods for analyses of CD4 count and treatment failure. To assess whether M184I/V was associated with VL failure independently of other mutations, we performed a separate analysis in which we used a mixed linear regression model adjusting for study as a random effect and other mutations associated with increased VL (which were identified by forward stepwise variable selection). Next, we used Fisher’s exact test to identify mutations associated with M184I/V. We used 2-sided *P* values and Stata version 15.1 for all statistical analyses.

### In Vitro Analyses

A patient-derived *pol* sequence was identified with mutations of interest, and the gag-PR-RT-IN region was amplified by polymerase chain reaction with flanking restriction sites inserted into primers. After cloning into an expression plasmid, site-directed mutagenesis was performed to revert isoleucine back to leucine at RT amino acid 74, valine back to methionine at RT amino acid 184, or both. Plasmids expressing gag-pol were cotransfected into 293T cells along with a vesicular stomatitis virus-G envelope expressing plasmid and a vector encoding luciferase expressed from a long terminal repeat promoter as previously described [42]. Supernatant containing virus was harvested 2 days later and used to infect fresh 293T cells. Luminescence as a read out of infection was read by luminometry 2 days later. Viral p24 abundance in supernatants was estimated using Western blot, using a p24 antibody as previously described [43].

## RESULTS

Among 2873 participants included in the initial group, 1445 from 32 study groups across 15 countries had an available failure VL measurement, and M184I/V was present in 817 (56.5%) of these (Table 1 and Supplementary Table 1). Participants were from sub-Saharan Africa (55.4%), Asia (19.2%), Europe (16.2%), and North America (9.3%). All participants were on TDF, most of them were also treated with EFV (75.2%) and 3TC (64.5%), and participants harboring M184I/V-mutated virus were significantly more likely to have high-level tenofovir and NNRTI resistance (Table 2). Participants harboring M184I/V were also more likely to have multiple NNRTI mutations.

**Table 1.** Baseline Characteristics of Participants by Geographic Region

Region	M184 I/V	Patients	EFV	3TC	Baseline CD4 Count		Baseline Log_10_ Viral Load	
					N With Data		N With Data	
Overall	No	628	523 (83.3%)	350 (55.7%)	351	180.0 (82.0 to 288.0)	253	5.0 (4.5 to 5.5)
	Yes	817	564 (69.0%)	**582 (71.2** **%)**	**385**	**88.0 (36.0 to 165.0)**	**187**	5.2 (4.7 to 5.7)
Sub-saharan Africa	No	257	198 (77.0%)	204 (79.4%)	142	148.0 (69.0 to 264.0)	43	5.3 (4.5 to 5.7)
	Yes	543	356 (65.6%)	430 (79.2%)	270	77.0 (35.0 to 138.0)	71	5.3 (4.7 to 5.7)
Asia	No	136	112 (82.4%)	110 (80.9%)	**0**	**-**	**0**	**-**
	Yes	**141**	**121 (85.8%)**	122 (86.5%)	4	69.5 (33.5 to 159.0)	5	4.7 (4.6 to 5.9)
Europe	No	146	127 (87.0%)	25 (17.1%)	138	199.5 (84.0 to 304.0)	136	5.0 (4.6 to 5.5)
	Yes	88	53 (60.2%)	23 (26.1%)	77	157.0 (62.0 to 232.0)	76	5.1 (4.8 to 5.7)
North America	**No**	**89**	**86 (96.6%)**	11 (12.4%)	71	204.0 (98.0 to 351.0)	77	4.7 (4.3 to 5.3)
	Yes	45	34 (75.6%)	7 (15.6%)	34	67.5 (27.0 to 156.0)	35	5.2 (4.8 to 5.6)

Abbreviations: 3TC, lamivudine; EFV, efavirenz.

**Table 2.** Summary of Drug Resistance Characteristics of Participants at Virological Failure With Tenofovir + Cytosine Analog + NNRTI by Geographical Region

Region	M184 I/V	TDF Resistance, n (%)	At Least One Major NNRTI Mutation, n (%)	Number of NNRTI Mutations, Mean (SD)	Failure Log_10_ Viral Load		Failure CD4 Count
						N With Data	Median (IQR)
Overall	No	137 (21.8%)	380 (60.5%)	1.2 (1.3)	4.3 (3.4 to 5.0)	237	263.0 (121.0 to 382.0)
	Yes	539 (66.0%)	792 (96.9%)	2.9 (1.3)	4.7 (4.1 to 5.3)	211	104.0 (29.0 to 236.0)
Sub-saharan Africa	No	80 (31.1%)	175 (68.1%)	1.5 (1.4)	4.7 (3.9 to 5.2)	29	262.0 (180.0 to 360.0)
	Yes	400 (73.7%)	531 (97.8%)	2.9 (1.3)	4.8 (4.1 to 5.3)	52	137.0 (20.0 to 219.0)
Asia	No	30 (22.1%)	91 (66.9%)	**1.3 (1.4**)	**4.8 (4.1 to 5.3)**	**119**	188.0 (71.0 to 355.0)
	Yes	82 (58.2%)	130 (92.2%)	2.9 (1.5)	4.9 (4.2 to 5.3)	118	87.5 (29.0 to 229.0)
Europe	No	20 (13.7%)	65 (44.5%)	**0.7 (1.0)**	**3.4 (2.7 to 4.6)**	**32**	323.0 (238.0 to 387.0)
	Yes	**38 (43.2%)**	**86 (97.7%)**	2.6 (1.4)	4.2 (3.8 to 4.8)	12	242.5 (122.0 to 345.0)
North America	No	7 (7.9%)	49 (55.1%)	0.8 (0.9)	3.4 (2.4 to 4.3)	57	312.0 (198.0 to 476.0)
	Yes	19 (42.2%)	45 (100.0%)	2.8 (1.4)	4.2 (3.7 to 4.7)	29	173.0 (42.0 to 329.0)

Abbreviations: IQR, interquartile range; NNRTI, nonnucleoside reverse-transcriptase inhibitor; SD, standard deviation; TDF, tenofovir disoproxil fumarate.

**Figure 1.**
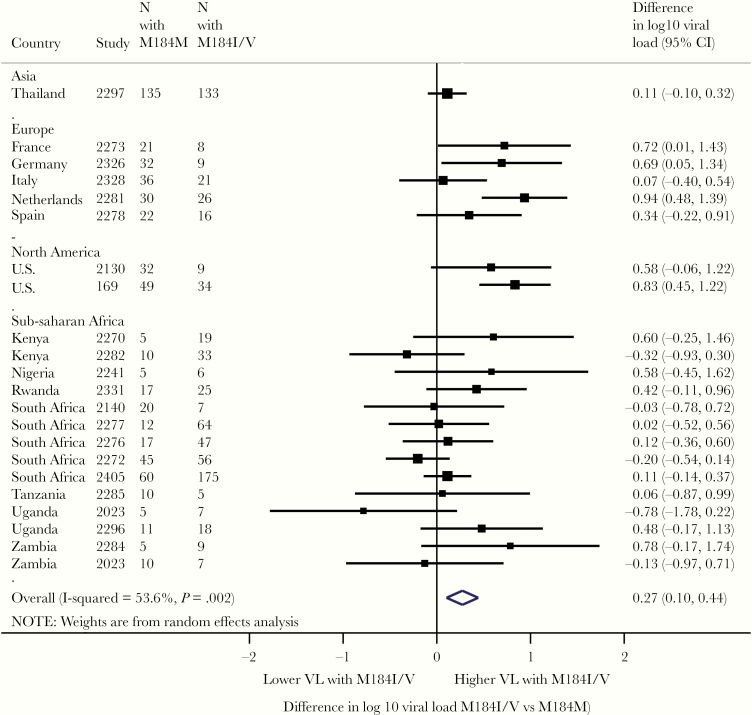
Difference in viral load by mutations at reverse-transcriptase position 184 in study groups with 95% confidence interval (CI) using random-effects meta-analysis. Boxes represent means, lines represent 95%. Estimates to the right indicate higher viral load in the presence of M184V/I, and estimates to the left indicate lower viral load in presence of M184V/I.

**Figure 2.**
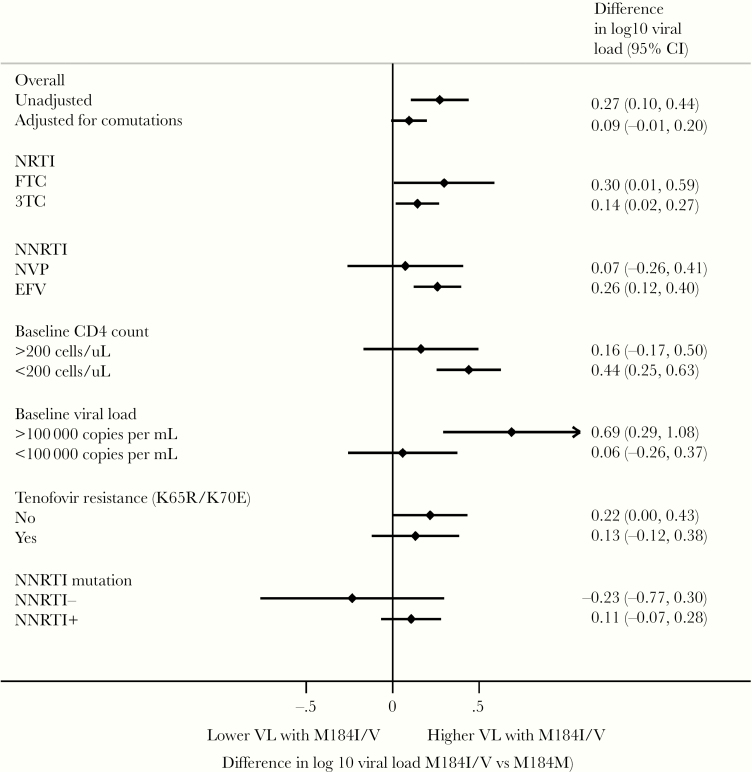
Association of M184V/I mutation with log_10_ viral load across subgroups. Diamonds represent means, lines represent 95%. Estimates to the right indicate higher viral load in the presence of M184V/I. 3TC, lamivudine; EFV, efavirenz; FTC, emtricitabine; NNRTI, nonnucleoside reverse-transcriptase inhibitor; NRTI, nucleoside reverse-transcriptase inhibitor; NVP, nevirapine.

In a crude comparison of VL failure, patients with M184I/V present had a higher median log_10_ VL (4.7; interquartile range [IQR], 3.4–5) than patients without M184I/V (median 4.3; IQR, 4.1–5.3). When restricting analyses to comparisons of patients within the same study, the estimated difference in VL was nonsignificant in the vast majority of studies (Figure 1). When within-study differences were pooled across studies, there was a marginally higher VL in patients with M184I/V present compared with absent (pooled difference in log_10_ VL, 0.18; 95% confidence interval [CI], .05–.31) (Figure 2). After statistical adjustment for other mutations independently associated with increased VL, M184I/V was no longer significantly associated with VL failure. However, the estimated difference and 95% CI (0.09; 95% CI, −.01 to .20) excluded any meaningful decrease in VL failure associated with M184I/V. There was no evidence that relationship between M184I/V and VL failure was modified by choice of NRTI, choice of NNRTI, or drug resistance to NNRTI or tenofovir (Figure 2).

**Figure 3.**
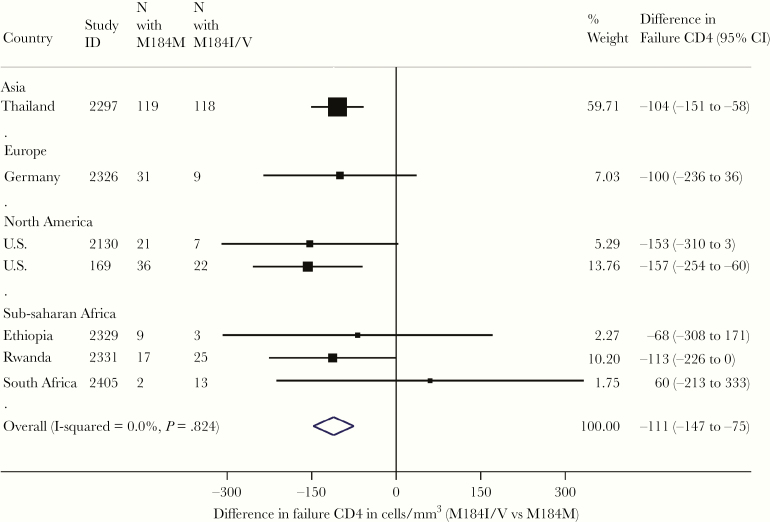
Differences in CD4 count during virological failure within studies by presence and absence of M184V/I. Boxes represent means, lines represent 95%. Estimates to the left of center line indicate lower CD4 count in participants with M184V/I.

We next explored the relationship between detection of M184I/V failure and CD4 count, noting that the duration of VF was likely longer in LMIC regions. Mean baseline CD4 was significantly lower among patients who went on to develop M184I/V by treatment failure compared with those who did not (88 vs 180, *P* < .0001). CD4 count at VF was also lower in patients with M184V/I than those without (Figure 3). Between baseline and treatment failure, CD4 count increased to a similar extent in patients with and without M184I/V (median increase, 79 vs 48 cells/mm^3^; *P* = .55).

**Figure 4.**
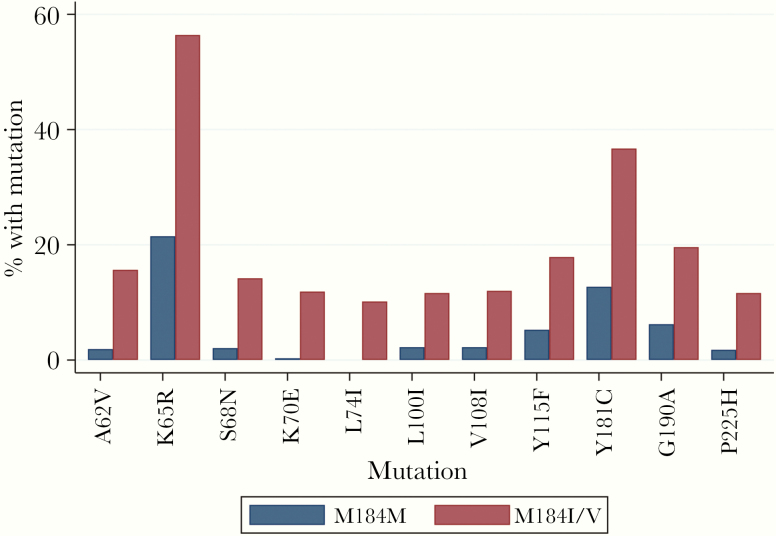
Human immunodeficiency virus reverse-transcriptase inhibitor resistance-associated mutations enriched in virologically failing participants (n = 1445) with M184V/I. Mutations are shown that occurred in at least 10% of individuals with M184V/ at a significance level of <.001.

We then examined NRTI mutations associated with M184V/I that might play a compensatory role for M184I/V. We looked for associations in the dataset between M184V/I and RT amino acid positions known to be associated with drug exposure. Figure 4 shows mutations with strong evidence of an association with M184I/V. Many of these mutations have previously been associated with drug resistance to tenofovir, either directly (K65R, K70E) or as compensatory mutations for K65R (A62V, S68N, F155Y). The following NNRTI mutations were also associated: A98G, L100I, K103R, V108I, Y181C, Y188L, G190A, P225H, L228R, and M230L.

Of note, L74I was the only mutation to be exclusively associated with M184V/I, occurring in 83 (10.2%) of patients with M184I/V, but not in 628 patients in which M184I/V was absent (*P* for association <.0001). L74I was observed in 11.7% of subtype C-infected participants with M184I/V at VF and in 14.4% of CRF01_AE participants with M184I/V at VF (Supplementary Table 2).

**Figure 5.**
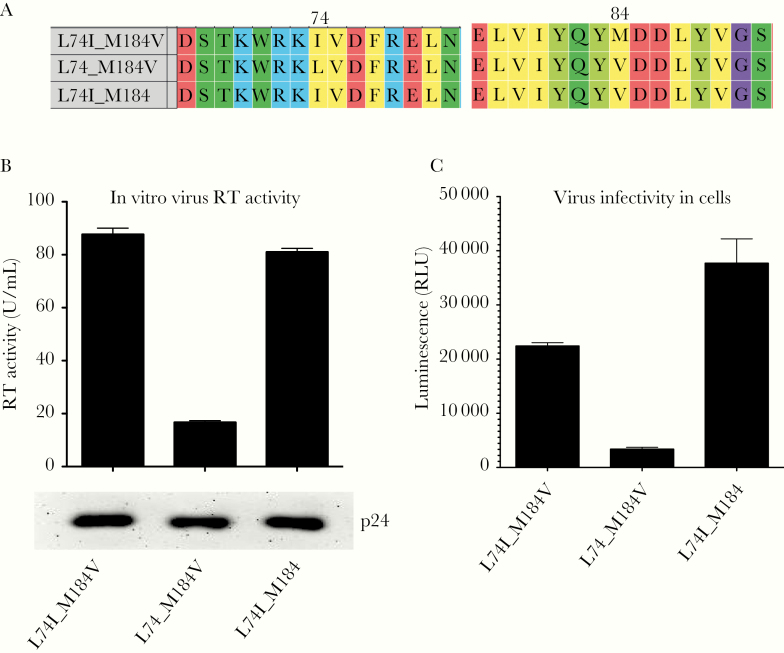
In vitro replication measurement of lamivudine-resistant subtype C clinical isolate containing M184V and L74I and revertant mutations. (A) Amino acid multiple sequence alignment of clinical isolate and revertant mutants generated by site-directed mutagenesis. Numbering is relative to strain HXB2. (B) In vitro reverse-transcription (RT) efficiency contained in pelleted single-round virus from cells producing clinical human immunodeficiency virus (HIV) isolate RT sequence and mutants. Bottom panel shows Western blot of corresponding virus-associated p24 in supernatants from cells. (C) Single-round infection of target HEK 293T cells by equal quantities of luciferase expressing vesicular stomatitis virus-G pseudotyped HIV viruses from B. Data in B and C were performed in replicate, and means are presented with error bars corresponding to standard deviation. RLU, relative light units.

A previous study reported that L74I can restore replication to a virus with the K65R mutation without conferring drug resistance [44]; therefore, we sought to test the hypothesis that L74I could restore replication “fitness” to a M184V mutant virus, thereby explaining the higher than expected VLs. Molecular characterization of virus with the mutations M184V and L74I was undertaken. The viral isolate tested also had NNRTI resistance mutations A98G, K103N, and P225H. Site-directed mutagenesis was performed to revert isoleucine back to leucine at 74 and revert valine to methionine at 184 (Figure 5A). However, we did not assess the impact of M184I. We measured (1) infectivity of these viruses and (2) RT efficiency in a single-round replication assay (Figure 5). We found that removing the L74I mutation significantly decreased the efficiency of reverse transcription (Figure 5B, compare left bar with middle bar), whereas virus abundance was not affected, as determined by Western blot of viral p24 abundance in supernatants (Figure 5B, bottom panel). Infectivity was also significantly decreased by reversion of the compensatory mutation (Figure 5C, compare left bar with middle bar). Mutation of M184V back to M, leaving a virus with only L74I, had no impact on RT efficiency and a minor effect on infectivity (Figure 5B and C, compare left and right bars).

## DISCUSSION

Despite having a low genetic barrier to drug resistance, 3TC has retained importance and a central role in both first- and second-line ART [45]. Therefore, a complete understanding of 3TC efficacy is important, particularly given reports suggesting that 3TC use confers VL benefit despite high-level resistance to the drug in the form of the M184V/I.

Our primary finding that VL was similar in participants with and without M184V/I at the time of VF was robust across baseline CD4 count, baseline VL, gender, and different NNRTI and NRTI drugs in the first-line treatment regimen. We observed lower baseline and VF CD4 counts in individuals with M184V/I, although rate of change of CD4 did not differ based on M184V/I status. Lower baseline CD4 count is known to be associated with higher VF rates and a higher probability of drug resistance at VF [6, 46]. A possible explanation for this finding is that the antiviral effect of a competent immune system is important in limiting replication and emergence of resistance in tissue compartments where ARV drug penetration is suboptimal. A lower CD4 count at VF in the group with M184V/I further argues against this mutation being “protective” or “benign.” These data are also consistent with reports of the pathogenic potential of M184V-containing viruses in both humans [47] and animal models [48].

We identified L74I as being specifically enriched in individuals with M184V and not present at all in those without M184V/I. We observed significant prevalence of L74I in subtypes C and CRF01_AE, although limited numbers of participants across subtypes hindered a full understanding of subtype distribution. In vitro experiments demonstrated that L74I restores replication efficiency to a virus with the M184V mutation over a single round of infection, and that enhancement was due to efficiency of HIV reverse transcription in viral particles.

The emergence of L74I exclusively in patients with M184V/I suggests an in vivo selection advantage of L74I + M184V replication over M184V alone, at least in some individuals. L74I was first reported as a mutation associated with exposure to abacavir or less commonly tenofovir [49, 50], and it appeared more common in patients with TAMs [50]. Correlation with M184V/I has not been made to date, and in vitro experiments have not been performed with L74I + M184V/I-containing viruses.

Because L74I was observed only in approximately 10% of those with M184V/I, we postulate that alternative mutations, less strongly linked to M184V/I or perhaps outside the region of the *pol* gene sequenced in this study, could have similar effects as L74I in participants with M184V/I. Data from our study support the transmission potential of M184V/I-containing viruses in the context of prolonged VF and accumulated coevolved mutations in RT that occurs under “real-world” conditions.

Limitations of this study include its retrospective cross-sectional design, absence of drug levels or adherence data, and unknown duration of VF for participants. Our study was not designed to provide a mechanistic understanding of the relationship between M184 and fitness: it was designed to understand the pathogenic potential of M184V-containing viruses in patients treated in the real world. Finally, there was heterogeneity between population groups, and, to account for this, analyses were conducted within study. It should also be noted that stratification by tenofovir or NNRTI resistance resulted in small numbers for subanalyses.

## CONCLUSIONS

In summary, we show that 3TC-resistant and 3TC-susceptible viruses show similar VLs in patients failing NNRTI-based ART containing 3TC, tenofovir, and NNRTI, likely in part due to viral evolution of compensatory changes that maintain replication efficiency of M184V/I-containing viruses. These data reinforce the importance of effective VL monitoring to limit HIV drug resistance and disease progression in the face of suboptimal drug pressure, particularly in low-resource settings. Finally, given that we did not find benefit of 3TC in patients failing first-line treatment, a prospective clinical trial could determine whether there is benefit for including XTC in second-line regimens for the treatment of persons whose viruses develop M184I/V after VF on a first-line treatment regimen.

## Supplementary Data

Supplementary materials are available at *The Journal of Infectious Diseases* online. Consisting of data provided by the authors to benefit the reader, the posted materials are not copyedited and are the sole responsibility of the authors, so questions or comments should be addressed to the corresponding author.

jiz631_suppl_Supplementary_Figure_1

jiz631_suppl_Supplementary_Table_1

jiz631_suppl_Supplementary_Table_2

## Notes

***Acknowledgments.*** We thank the participants. We also thank Petra Mlcochova for assistance with Western blotting.

***Financial support.*** This study was funded by the Wellcome Trust.

***Potential conflicts of interest*.** R. K. G. has acted as ad hoc consultant for Gilead Sciences and ViiV. C. F. P. has acted as ad hoc consulant for Gilead Sciences, Merck, Janssen, Theratechnologies, and ViiV. R. S. S. has received research funding from Janssen. All authors have submitted the ICMJE Form for Disclosure of Potential Conflicts of Interest. Conflicts that the editors consider relevant to the content of the manuscript have been disclosed.
