# Supplementary material for: Human Immunodeficiency Virus-1 Viral Load Is Elevated in Individuals With Reverse-Transcriptase Mutation M184V/I During Virological Failure of First-Line Antiretroviral Therapy and Is Associated With Compensatory Mutation L74I
Source: J Infect Dis. 2019 Nov 27;222(7):1108–16. doi: 10.1093/infdis/jiz631 (PMC7459140; doi:10.1093/infdis/jiz631)
Supplement: jiz631_suppl_Supplementary_Figure_1 [file jiz631_suppl_supplementary_figure_1.docx]

Supplementary Figure 1: Difference in viral load at virological failure in the presence of M184I (left panel) or M184V (right panel) versus M184M within study groups with 95% confidence interval using random effects meta-analysis. Boxes represent mean with 95% CI.
