# Supplementary material for: Human Immunodeficiency Virus-1 Viral Load Is Elevated in Individuals With Reverse-Transcriptase Mutation M184V/I During Virological Failure of First-Line Antiretroviral Therapy and Is Associated With Compensatory Mutation L74I
Source: J Infect Dis. 2019 Nov 27;222(7):1108–16. doi: 10.1093/infdis/jiz631 (PMC7459140; doi:10.1093/infdis/jiz631)
Supplement: jiz631_suppl_Supplementary_Table_1 [file jiz631_suppl_supplementary_table_1.docx]

**Supplementary Table 1.** List of studies/datasets with publicly available RT sequences from individuals failing a TDF+XTC+NNRTI-containing first-line regimen

| **Author** | **PubMedID** | **Number of individuals** | **Regions** |
| --- | --- | --- | --- |
| ***TenoRes Studies***  (29 datasets, 1573 individuals) | | | |
| Sirivichayakul, S |  | 282 | Thailand |
| Theys, K | 23183438 | 203 | Belgium, Germany, Israel, Italy, Luxembourg, Portugal, Spain, Sweden |
| Stanford, A | 28365230 | 118 | U.S. |
| Hunt, G | 28981637 | 115 | South Africa |
| Goedhals, D |  | 102 | South Africa |
| de Oliveira, T |  | 81 | South Africa |
| Rokx, C | 25273080 | 68 | Netherlands |
| Santoro, M |  | 65 | Italy |
| Yang, C |  | 56 | Kenya |
| Schmidt, D |  | 53 | Germany |
| Hoffmann, CJ | 23751421 | 50 | South Africa |
| Sobrino-Vegas, P | 21820763 | 40 | Spain |
| Neogi, U | 24922326 | 38 | Sweden |
| Kaleebu, P | 26700639 | 35 | Uganda |
| Brooks, K | 27231099 | 32 | Kenya |
| Sunpath, H | 22739389 | 31 | South Africa |
| Charpentier, C |  | 31 | France |
| Theys, K | 23027713 | 30 | Portugal |
| Etiebet, MA | 23079810 | 21 | Nigeria |
| Kerschberger, B |  | 21 | Swaziland |
| Yang, C |  | 17 | Zambia |
| Yang, C |  | 15 | Tanzania |
| Shapiro, J |  | 14 | Israel |
| Arruda, M |  | 13 | Brazil |
| Ndembi, N |  | 12 | Nigeria |
| Yang, WL | 26362944 | 10 | Switzerland |
| Ugbena, R | 22544206 | 7 | Nigeria |
| Hamers, RL |  | 7 | Nigeria, Uganda, Zambia |
| Yang, C |  | 6 | Uganda |
| ***Non-TenoRes Studies***  (22 datasets, 1840 individuals) | | | |
| Van Zyl, GU |  | 466 | South Africa |
| Steegen, K | 27659733 | 322 | South Africa |
| Van Zyl, GU | 23840622 | 151 | South Africa |
| Neogi, U | 26413747 | 146 | South Africa |
| Dinesha, TR | 27334566 | 144 | India |
| Theys, K |  | 121 | Belgium, Germany, Italy, Luxembourg, Portugal, Sweden |
| Lam, EP | 27346600 | 102 | Argentina, India, Israel, Malaysia, Mexico, Nigeria, South Africa, Thailand, U.K. |
| Skhosana, L | 25659108 | 79 | South Africa |
| Ndahimana, JD | 27125473 | 68 | Rwanda |
| Hamers, RL | 22474222 | 47 | Nigeria, South Africa, Uganda, Zambia, Zimbabwe |
| Mollan, K | 23148287 | 44 | U.S. |
| Hawkins, CA | 19644383 | 24 | Nigeria |
| Sigaloff, KC | 21694603 | 21 | Kenya, Nigeria, South Africa, Uganda, Zambia |
| Ngo-Giang-Huong, N | 22132100 | 19 | Thailand |
| Seu, L | 25754408 | 19 | Zambia |
| Jiamsakul, A | 25141905 | 15 | Philippines, Thailand |
| Riddler, SA | 18480202 | 12 | U.S. |
| Abdissa, A | 24708645 | 12 | Ethiopia |
| Rey, D | 19036752 | 8 | France |
| Avidor, B | 23469241 | 8 | Israel |
| Non-B Workgroup | 15839752 | 7 | Portugal, U.K. |
| Khairunisa, SQ | 25348045 | 5 | Indonesia |
| TDF - tenofovir disoproxil fumarate; TenoRes Studies – studies included in the TenoRes analysis (TenoRes Study Group, Lancet Infect Dis. 2016). | | | |
