# Supplementary material for: Human Immunodeficiency Virus-1 Viral Load Is Elevated in Individuals With Reverse-Transcriptase Mutation M184V/I During Virological Failure of First-Line Antiretroviral Therapy and Is Associated With Compensatory Mutation L74I
Source: J Infect Dis. 2019 Nov 27;222(7):1108–16. doi: 10.1093/infdis/jiz631 (PMC7459140; doi:10.1093/infdis/jiz631)
Supplement: jiz631_suppl_Supplementary_Table_2 [file jiz631_suppl_supplementary_table_2.docx]

| SUBTYPE | L74L | L74I | L74V |
| --- | --- | --- | --- |
| A | 42 (95.5%) | 1 (2.3%) | 1 (2.3%) |
| B | 107 (89.2%) | 9 (7.5%) | 4 (3.3%) |
| C | 349 (83.3%) | 49 (11.7%) | 21 (5.0%) |
| CRF01_AE | 115 (82.7%) | 20 (14.4%) | 4 (2.9%) |
| CRF02_AG | 18 (100.0%) | 0 (0%) | 0 (0%) |
| D | 36 (94.7%) | 1 (2.6%) | 1 (2.6%) |
| F | 3 (100.0%) | 0 (0%) | 0 (0%) |
| G | 31 (93.9%) | 1 (3.0%) | 1 (3.0%) |
| K | 3 (100.0%) | 0 (0%) | 0 (0%) |

Supplementary table 1: Subtype distribution of mutations at RT position 74 in participants with RT M184V/I detected by Sanger sequencing at virological failure.
